# Snapshot: a package for clustering and visualizing epigenetic history during cell differentiation

**DOI:** 10.1186/s12859-023-05223-1

**Published:** 2023-03-20

**Authors:** Guanjue Xiang, Belinda Giardine, Lin An, Chen Sun, Cheryl A. Keller, Elisabeth F. Heuston, Stacie M. Anderson, Martha Kirby, David Bodine, Yu Zhang, Ross C. Hardison

**Affiliations:** 1 The Bioinformatics and Genomics Program, Huck Institutes of the Life Sciences, The Pennsylvania State University, University Park, PA USA; 2 Department of Biochemistry and Molecular Biology, The Pennsylvania State University, University Park, PA USA; 3 Department of Computer Science and Engineering, The Pennsylvania State University, University Park, PA USA; 4 NHGRI Hematopoiesis Section, GMBB, Bethesda, MD USA; 5 NHGRI Flow Cytometry Core, Bethesda, MD USA; 6 Department of Statistics, The Pennsylvania State University, University Park, PA USA

**Keywords:** cCRE indexing, cCRE Clustering and Visualization, Epigenetic state visualization, Cell differentiation

## Abstract

**Background:**

Epigenetic modification of chromatin plays a pivotal role in regulating gene expression during cell differentiation. The scale and complexity of epigenetic data pose significant challenges for biologists to identify the regulatory events controlling cell differentiation.

**Results:**

To reduce the complexity, we developed a package, called Snapshot, for clustering and visualizing candidate cis-regulatory elements (cCREs) based on their epigenetic signals during cell differentiation. This package first introduces a binarized indexing strategy for clustering the cCREs. It then provides a series of easily interpretable figures for visualizing the signal and epigenetic state patterns of the cCREs clusters during the cell differentiation. It can also use different hierarchies of cell types to highlight the epigenetic history specific to any particular cell lineage. We demonstrate the utility of Snapshot using data from a consortium project for **V**al**I**dated **S**ystematic **I**ntegrati**ON** (VISION) of epigenomic data in hematopoiesis.

**Conclusion:**

The package Snapshot can identify all distinct clusters of genomic locations with unique epigenetic signal patterns during cell differentiation. It outperforms other methods in terms of interpreting and reproducing the identified cCREs clusters. The package of Snapshot is available at GitHub: https://github.com/guanjue/Snapshot.

## Background

The gene regulation community has generated thousands of epigenomic datasets, and integration of these data has become a powerful step in facilitating studies to better understand the biological meaning of combinations of epigenetic events [2–7]. Experiments such as ATAC-seq and DNase-seq, which measure the accessibility of genomic regions in chromatin [8–10], have been widely used to identify candidate cis-regulatory elements (cCREs). The cCREs are often defined as having a strong peak-like signals for ATAC-seq or DNase-seq in one or multiple cell types, indicating these DNA segments are more exposed in chromatin. This greater accessibility of the DNA may result from nucleosome destabilization and transcription factor binding, and hence these DNA segments may be inferred to have potential function on regulating proximal and/or distal genes [5, 6, 10]. Additional information about the potential activity of cCREs in a cell type can come from data on histone modifications and other epigenetic features in the cCREs and surrounding chromatin. This information can be concisely summarized by learning the unique combinations of epigenetic features that frequently occur in chromatin, which are referred to as epigenetic states [11–13]. Annotating cCREs by their accessibility and/or their epigenetic states across a series of cell types can enhance our understanding of the roles they play in gene regulation [5, 7].

One common analysis of cCREs compares the intensity of specific epigenomic signals between two cell types. The cCREs that exhibit differential patterns can provide insights into gene regulation mechanisms, such as identifying cell type-specific transcription factors operating at the cCREs [14, 15]. As more sets of epigenomic data are generated, it has become common to cluster and analyze patterns of epigenomic signal at cCREs across multiple cell types. For example, clustering cCREs based on their DNase-seq signal across multiple cell types can reveal both cell type-specific actuation of cCREs and cCREs with more complex functions within different groups of cell types [10]. Furthermore, methods have been developed to infer the epigenetic states at cCREs more accurately by borrowing information across multiple cell types [11] or by leveraging information from multiple cell types to correct potential false differential epigenomic calls [16].

Clustering candidate cCREs based on their presence or absence or based on signal intensity across multiple cell types is a commonly used approach to uncover activity patterns of cCREs, and hence their potential regulatory function, across various cell types [17, 18]. For example, the distance-based methods such as K-means and hierarchical clustering can group the cCREs into different categories based on their chromatin accessibility signals across multiple cell types [19–21]. However, these methods implicitly assume that the signals of cCREs in different cell types are independent from each other, which is problematic because some cell types are related by the process of cell differentiation. To account for the association of cCRE signals, some model-based methods treat the signals of cCREs across multiple cell types as multivariate observations [22]. The covariance of the multivariate observations can be used to capture the signal associations. Some methods treat the cell types along a cell differentiation lineage as a time series and use Gaussian process mixture model to cluster cCREs [23]. Several methods further use either infinite Gaussian mixture models or Dirichlet processes to automatically determine the number of the clusters [24–26]. However, these model-based methods tend to create large clusters of cCREs, while smaller but unique cCRE clusters are often lost by being merged into the larger ones. Furthermore, these methods do not consider any existing biological knowledge about the cell type relationships. As a result, interpreting the biological meaning of the identified cCRE clusters can be difficult and irreproducible, especially when the number of cell types is large. In addition, for some of methods, such as the method using Gaussian process mixture model, the computational costs can be high for large datasets [23]. These clustering methods find informative groups of discrete genomic elements, such as cCREs, that are not contiguous in the genome. Such clustering results can be complemented by different unsupervised methods examining contiguous epigenomic signals, such as ChromHMM running in the stacked modeling mode [27]. The latter approach can find epigenetic states that are restricted to certain cell types as well as states found in all examined cells, which could correspond to some of the groups of cCREs identified by clustering methods.

**Fig. 1:**
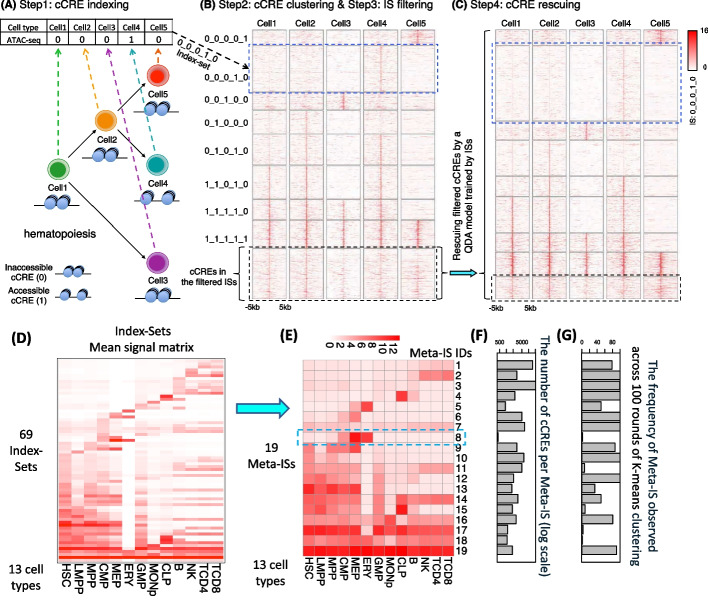
Overview of Snapshot. **A** Step1: cCRE indexing. A binarized index is created for each cCRE based on the presence/absence pattern of the cCRE across all cell types. **B** Step2: cCRE clustering and Step3: filtering. The cCREs with the same index were clustered into an Index-Set (IS). For example, the cCREs with 0_0_0_1_0 index were clustered into the IS in blue dash box. The cCREs in the less abundant ISs, highlighted by a black dash box, were filtered. **C** Step4: cCREs rescuing. The cCREs in the filtered ISs were re-classified as members of the abundant ISs based on their posterior probabilities of multivariate Gaussian distributions (using a Quadratic Discriminant Analysis (QDA) model) of the abundant ISs. The heatmaps in panels B and C were generated by deeptools [50]. **D** The mean signal matrix for all 68 abundant Index-Sets and an additional Index-Set, which included all remaining cCREs not assigned to an abundant IS. **E** The cCRE mean signal heatmap for the 19 Meta-Index-Sets (Meta-ISs) merged from 69 ISs. The number of Meta-ISs are automatically determined by AIC. **F** The bar plot for the number of cCRE within each Meta-ISs in log scale. **G** The frequency at which the signal pattern of a of Meta-IS was observed in 100 rounds of K-means clustering

Here we present a package, called Snapshot, for clustering and visualizing the cCREs and their epigenetic states during cell differentiation. The package uses a binarized indexing strategy for grouping the cCREs into different clusters (Fig. 1). The strategy will identify all binarized cCRE clusters in the data, and it further merges them into interpretable groups. It automatically determines the number of clusters to analyze. Furthermore, the clusters and the corresponding dominant epigenetic states in each of the cell types can be visualized by incorporating a user provided cell differentiation tree, and thus can highlight the epigenetic history specific to any particular cell lineage. In this paper, we used the data generated by the VISION project [5, 28–30] to demonstrate the improved performance of Snapshot over existing methods in terms of interpretability, comprehensiveness, and robustness of understanding the biological functions of the hematopoietic cCRE clusters.

## Implementation

### Description of the snapshot *package*

**Fig. 2:**
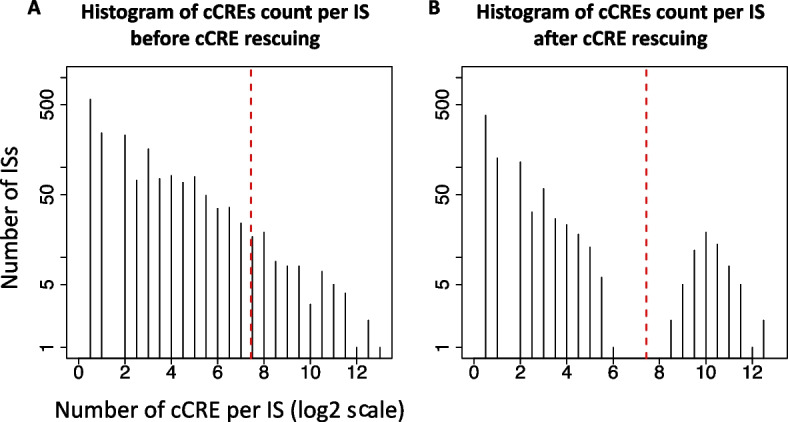
Distribution of cCRE count per IS before **A** and after **B** rescuing cCREs. In these histograms, the number of ISs on the y-axis is shown on a log (base 10) scale, and the number of cCREs per IS is shown on a log (base 2) scale. The red dashed lines indicate the cCRE threshold (173) for abundant IS, which is determined based on FDR adjusted p-value (< 1e-2) calculated using a negative binomial model for the count of cCREs in each IS

This sub-section presents an overview of the Snapshot package, and subsequent sub-sections explain specifics of individual steps and components. The first step of Snapshot is to cluster the cCREs across cell types, e.g. across hematopoietic cell differentiation for the datasets examined here. To capture all distinct and abundant clusters, we first use a binarized index to encode the signals of each cCRE across multiple cell types (Step1: cCRE indexing, Fig. 1A). All the cCREs with the same index are assigned to the same initial cluster (Step2: cCRE clustering, Fig. 1B). We name each cluster an Index-Set (IS), and the size of the IS is defined as the number of cCREs in it. The first two steps can produce a large number of ISs that only have a few cCREs (Fig. 2A). We hypothesize that those ISs are minor variations of the abundant ISs or spurious ISs resulting from peak calling errors, and thus the cCREs within them should be re-classified into the abundant ISs. Thus, we introduce a filtering step followed by a rescuing step to achieve those goals. We first filter (temporarily) any ISs that are smaller than a size threshold determined automatically (or specified by the user) (Step3: IS filtering, Fig. 1B). We then fit the signals of the cCREs in each of the remaining, abundant ISs to a multivariate Gaussian distribution (MVN). Then, the fitted MVNs are used as prior distributions to re-classify the cCREs inside the filtered ISs, which adds many of cCREs that were in small initial ISs to larger ISs (Step4: cCRE rescuing, Fig. 1C). All the cCREs that have a posterior probability less than 0.5 were put into one set as a null cluster. This filtering procedure followed by rescue can not only greatly reduce the number of ISs, but it also can correct the potential errors in peak calling results by replacing the original indices of some cCREs by the indices of their newly assigned ISs. The ISs that result from the filtering and rescue are one output of the Snapshot package, e.g. the package placed the 83,701 human blood cell cCREs into 69 ISs (Fig. 1D). For some analyses, a smaller number of clusters may be desirable, and thus we added a merging step, using hierarchical clustering of the mean signal vectors of ISs, to further group the ISs into Meta-Index Sets (Meta-ISs), which comprise an additional output from the Snapshot package. For example, the 69 ISs for the VISION cCREs were combined into 19 Meta-ISs (Fig. 1E). The Snapshot package also generates a set of figures to visualize the average cCRE signals and the abundant epigenetic states across multiple cell types during cell differentiation for each IS and each Meta-IS. These visualizations are shown in subsequent sub-sections.

### *cCREs* indexing and *cCRE* clustering

The motivation for developing Snapshot arose from our observation that conventional clustering methods did not bring out important but small cCRE clusters. We reasoned that an indexing strategy would be guaranteed to capture all distinct clusters of cCREs. Our goal is to identify clusters of cCREs such that each represents a unique pattern of presence and absence calls of cCREs, which in turn can be inferred to represent a common potential gene regulatory function. For the first step in Snapshot, we use the binarized presence/absence status of chromatin accessibility peak calls across all cell types to create a cCRE index to represent the unique pattern. The number of bits in the index equals the number of cell types. The order of bits is the order of cell types derived from a user-provided cell differentiation tree. The order of the bits can be shifted by the user to focus on different aspects of the series of cell types. The indices readily group the cCREs into distinct clusters by assigning the ones with the same index to the same cluster. We define each of the clusters as an index-set (IS).

### IS filtering

Simply clustering on the indices can generate such a large number of ISs that the results are difficult to interpret biologically. We conduct a filtering step to restrict the ISs to those whose size exceeds an abundance threshold (Fig. 1B). The next sub-section describes a rescue procedure to re-assign the filtered cCREs to closely matching, larger ISs. In a system with N cell types, there can be 2^N possible ISs. In practice, we observed a large number of ISs, but most of them contain only a few cCREs (Fig. 2A). The filtering step in Snapshot temporarily removes all ISs whose size is smaller than an abundance threshold, which can be provided by the user or determined automatically in Snapshot by assuming a negative binomial (NB) background. To do so, we first fit a NB background model based on the sizes of initial ISs. When fitting the NB model, the most abundant ISs (top 5%) were excluded to avoid bias from outliers. We then compute the FDR adjusted p-values for the sizes of all ISs based on the NB background model. The size corresponding to an adjusted p-value of 0.01 was used as the abundance threshold. The clustering results were robust to changes in these thresholds; specifically, similar results were obtained after varying the percentage of most abundant ISs filtered from 2.5% to 10% and varying the adjusted p-value between 0.001 and 0.2.

### cCRE rescuing

The cCREs in the filtered, smaller sized ISs were then rescued by adding them back to the closest matching, larger sized ISs. These smaller sized ISs may consist of cCREs that have spurious peak calls resulting from noise in the chromatin accessibility data in one or a few cell types. Thus, we hypothesize that many of the smaller sized ISs are minor variations of the larger ISs, separating from the larger ISs because of cCREs with spurious peak calls. Even so, we can still assume that the peak calling results for these cCREs are accurate in most cell types, and matching the cCREs in the filtered ISs could be used to correct erroneous peak calling results in other cell types. Therefore, we developed a rescuing strategy to re-classify the filtered cCREs to one of the abundant ISs. To do so, we assume the epigenetic signals of cCREs across cell types in each IS follow one multivariate Gaussian distribution (MVN). Inside the filtered ISs, the cCREs’ posterior probabilities for these MVNs can be calculated to re-classify them into one of the abundant ISs (Fig. 1C). Specifically, we use all the abundant ISs remaining after filtering to train the Quadratic Discriminant Analysis (QDA) model [31]. Then, we use the trained model to re-classify each filtered cCREs to an abundant IS based on posterior probabilities across all abundant ISs. Let $\mathrm{x}$ denote the binary signal vector of each cCRE across cell types. The posterior probabilities ${\mathrm{P}}_{i}\left(\mathrm{x}\right)$ of a cCRE for the i-th IS is calculated by:

$$P_{i} (x) = - \frac{1}{2}log\left|\sum_{{\text{i}}} \right| - \frac{1}{2}(x -\upmu _{i} ){^{\prime}}\sum_{i}^{( - 1)} (x -\upmu _{i} ) + logP0_{{\text{i}}} ,$$

where ${\upmu }_{\mathrm{i}}$ and ${\Sigma }_{\mathrm{i}}$ denotes the mean vector and the covariance matrix of the i-th IS, and ${\mathrm{P}0}_{\mathrm{i}}$ denotes the proportion of cCREs in the i-th IS. The model will assign the cCRE to the IS with the highest posterior probabilities. The cCREs with the highest posterior probabilities less than 0.5 are assigned into a null class. Thus, all cCREs in the filtered ISs are re-assigned to either an abundant IS or the null IS. For each rescued cCRE, the initial index is replaced by the index of the abundant IS to which they were re-classified. This replacement can help correct any erroneous peak calling results for the cCRE in some cell types.

### Merge ISs into *meta-ISs*

For some applications, a smaller number of groups of cCREs could improve interpretability, so we implemented a second round of clustering to group the ISs with a similar mean signal vector into Meta-Index-Sets (Meta-IS). Here, the rationale is that the Snapshot index-based strategy can identify all cluster patterns, including those that are rare but important, but some of the ISs showed similar patterns (Fig. 1D). The second round of clustering utilizes the mean signal vector of each IS as the basis for clustering, which removes any dependency on the number of cCREs within each IS. For example, the relatively small ISs with erythroid cCREs are retained as Meta-IS 8 (Fig. 1F and G). This approach reduces the likelihood of missing rare but important cluster patterns, whereas cluster center initialization may miss these patterns due to their rarity. The merging into Meta-ISs uses the hclust R function followed by cutree R function. The number of clusters in the cutree function is determined based on the Akaike information criterion (AIC) [32].

### Optional data normalization within *snapshot*

The Snapshot package expects normalized input signals to reduce the influence of technical variations in signal scaling or signal-to-noise ratio on the clustering. However, many public datasets are not normalized, which can complicate the clustering and interpretation of results. To address this issue, we included several optional internal normalization methods, including scaling, quantile normalization, and S3norm [33]. For S3norm, Snapshot can first identify the IS containing cCREs that are common peaks across all cell types (common-peak-IS) and the IS containing cCREs that are in common background regions across all cell types (common-background-IS). It then adjusts the signal-to-noise ratio by scaling each dataset to an average reference signal-to-noise based on the mean signal difference between the common-peak-IS and common-background-IS.

### Assigning epigenetic states to cCREs, ISs, and meta-ISs

Many of the visualizations from the Snapshot package utilize the annotations of cCREs by their epigenetic states. Such annotations are often used to infer potential functions of each cCRE [5]. In Snapshot, we use bedtools [34] to assign an epigenetic state to each cCRE in each cell type. Since each 200 bp bin was annotated with one epigenetic state in each cell type, one cCRE that covers more than 200 bp genomic regions can simultaneously intersect with multiple 200 bp bins with different epigenetic states. For many downstream analyses, it is desirable to assign a single, dominant epigenetic state to each cCRE in each cell type. We systematically assign the single state using the following criteria. First, if a cCRE intersects with a non-quiescent state, it will not be assigned with quiescent state, i.e. one with undetectable signal for all epigenetic features examined. Second, when a cCRE intersects with multiple non-quiescent states, the state that covers the largest proportion of the cCRE region is assigned to the cCRE. Third, when a cCRE intersects with multiple non-quiescent states that cover the same proportion of the cCRE region, the state with a midpoint closest to the cCRE midpoint will be assigned to the cCRE. Fourth, when a cCRE intersect with multiple non-quiescent states that cover same proportion of the cCRE region and their midpoints to the cCRE midpoints are the same, the state that covers more base-pairs on the cCRE will be assigned to the cCRE. In practice, we have found that those four rules sufficed to assign a single epigenetic state each cCRE in a large collection, such as those in the VISION project for blood cells [5]. After assigning epigenetic states to all cCREs across all cell types, the Snapshot algorithm uses the most prevalent epigenetic states (those that cumulatively covering more than 50% of the cCREs in the IS or Meta-IS) as the representative state for each cell type in each IS or Meta-IS. One Snapshot output is a cell differentiation tree for each IS or Meta-IS, with each cell type colored by a summary of the representative epigenetic states. The color assigned to each cell type is determined as the weighted average of these representative states, with the weight being the proportion of cCREs in the IS or Meta-IS that are assigned to a representative state.

### Snapshot visualization module

**Fig. 3:**
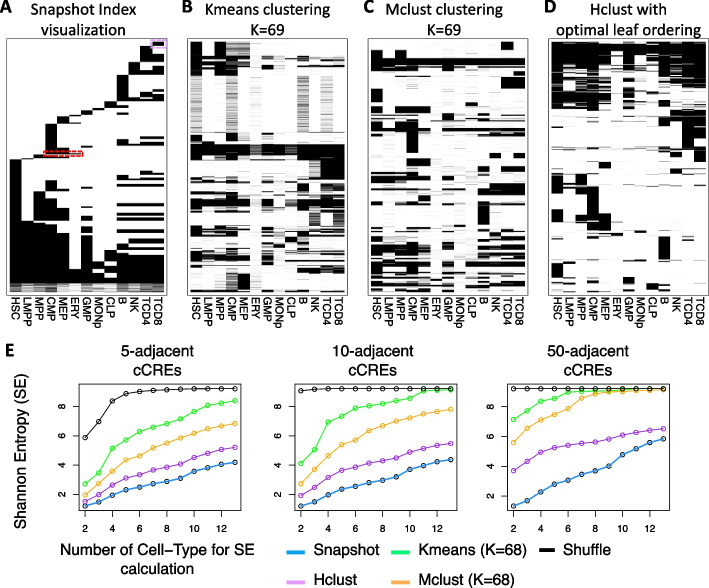
Comparison between the Snapshot IS clustering method and other existing clustering methods. **A** The binary map of the presence (black) or absence (white) pattern of cCREs across 13 cell types. Each row represents a cCRE, which has been ordered by the indices identified by Snapshot. The binary maps or results from clustering by K-means, Mclust, and the Hierarchical method are shown in panel **B-D**. **E** The Shannon Entropy (SE) of the binary map. The y-axis is the SE value. The x-axis represents the number of cell types used to calculate the SE. The 3 figures are shown the result using different settings for the height of scanning window (5, 10, or 50 adjacent cCRE at each step of scanning) in the SE calculation

Snapshot provides a set of visualizations to show various aspects of the ISs and their epigenetic features. One output is a collection of maps showing the binary patterns (e.g., Fig. 3A), a heatmap for the average ATAC-seq/DNase-seq signals (e.g., Fig. 1D and E), and a heatmap for the representative functional epigenetic state in each of the cell types in each of the ISs. A second output contains cell differentiation trees colored by either the average ATAC-seq/DNase-seq signals or the representative functional epigenetic states for each of the ISs. A third visualization provides bar-plots for the proportions of all epigenetic states in each of the cell type for each of the ISs. A fourth visualization has violin-plots for the ATAC-seq/DNase-seq signal distributions in each of the cell type for each of the ISs.

### Inputs for *snapshot*

Snapshot takes the following files as input: (1) peak calling results of epigenetic features in bed format [35]; (2) signal strength of the epigenetic feature across the whole genome in bigWig format; (3) functional epigenetic state labels in bigBed format; (4) a list of colors for each functional epigenetic state; and (5) a pairwise cell type relationship in cell differentiation tree. In addition, there is an option to provide a Master peak list of cCREs or other epigenetic features. Genome-wide data on any epigenetic feature (epigenomic data) can be used as input to Snapshot, as long as the epigenomic datasets have peak calls and signal tracks. The epigenetic features include DNase-seq, ATAC-seq, ChIP-seq for histone modifications and transcription factors, and DNA methylation. Furthermore, transcriptomic data can be used as inputs to study gene expression patterns across cell types.

### Evaluating interpretability of clustering results

The purpose of unsupervised clustering is to identify the de novo patterns in the data in an unbiased manner. Due to the high complexity of epigenetic signals across multiple cell types, the utility of the results is related to the interpretability of the de novo patterns in the clustering results. To quantify the interpretability of various clustering results, we employed the Shannon Entropy (SE) [36] as a metric. Our reasoning is that a more random clustering result is more difficult to interpret as it is less clear how the data points are grouped together. Conversely, if a user can easily understand the formation of each cluster, such as through the identification of active or inactive patterns in a specific group of related cell types, we believe the clustering result can be more easily interpreted and serve as a foundation for generating new ideas. Following this rationale, we used the SE to estimate the randomness of the clustering results obtained from various methods. A lower SE indicates that the clustering result is less random and therefore more likely to be interpretable. Thus, the SE provides a metric to quantitatively compare the interpretability of different clustering methods.

**Fig. 4:**
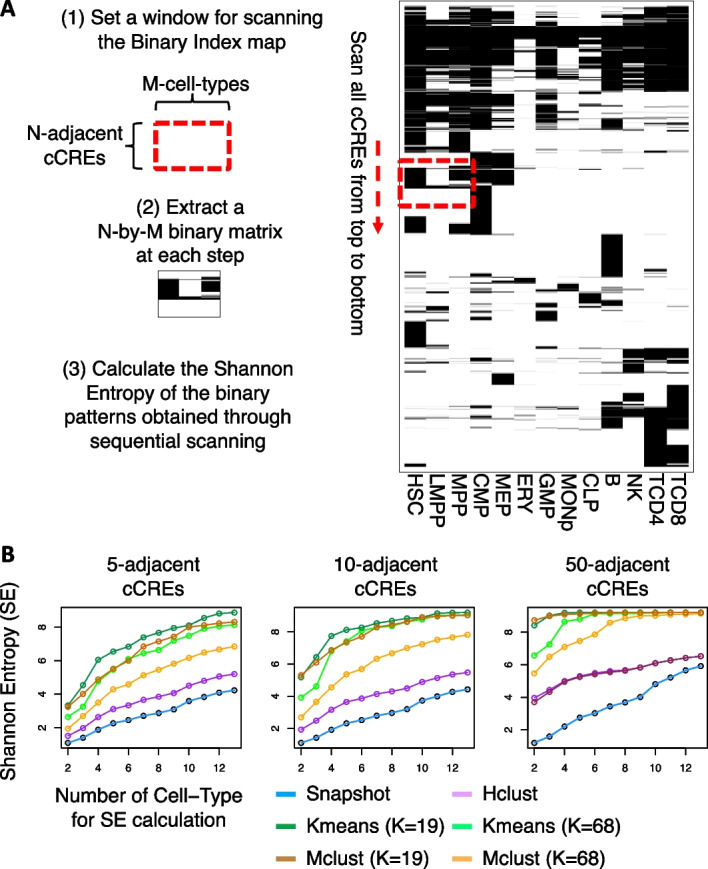
Illustration of the process for using Shannon Entropy (SE) to assess the interpretability of various clustering results. Panel **A** outlines the three key steps for the SE calculation. **B** The comparison of SE values obtained from Snapshot and other clustering methods, using different N-adjacent cCREs scanning windows for SE calculation, including comparisons with fewer clusters (K = 19)

In the specific procedure employed here (Fig. 4), the first step is to establish a 2-dimensional (2D) window for scanning and extracting local patterns in a binary index map. This window has a fixed height of N adjacent cCREs and a width of M cell types, which define the local region for each scanning step. The second step slides the 2D window one cCRE at a time to scan to binary index map from top to bottom. The sliding window works like the convolutional layer in convolutional neural network [37]. At each step, a N-by-M binary pattern is extracted. In the third step, we calculate the SE using the count of each unique N-by-M binary pattern generated from the scanning process. We calculate the probability of each unique N-by-M binary pattern by dividing the number of its occurrences by the total number of scanning steps. This probability was used as the ${\mathrm{P}}_{i}$ for the following SE formula:

$${\text{SE}} = - \mathop \sum \limits_{{\text{i}}} {\text{P}}_{{\text{i}}} \ln {\text{P}}_{{\text{i}}} ,$$

where i denotes the i-th unique N-by-M binary pattern. We further calculate the SE for larger local regions by increasing the number of cell types (M cell types, where M ranges from 2 to 13) in the sliding window. This allows us to evaluate the interpretability of each clustering method when focusing on different subsets of cell types in the results.

### Evaluating performance of K-means clustering in identifying rare clusters

To evaluate the performance of the K-means clustering method in identifying rare cCRE clusters (Fig. 1G), we performed 100 rounds of K-means (K = 19) clustering on the same data matrix with different random seeds. For each round, we calculated the cosine distance between the mean signal vectors of all K-means clusters and the mean signal vectors of all Snapshot Meta-IS clusters. If at least one K-means cluster was closest to a Meta-IS, we add one to the number of times the Meta-IS appearing in the 100 rounds of K-means clustering.

### Preparing a master peak list across multiple cell type

In the Snapshot package, users have the option to either provide their own master peak list for analysis or utilize Snapshot's built-in function to generate a master peak list from the input peak bed files. In the latter case, Snapshot will concatenate the peaks in all cell types into one peak file, and then merge those with at least 1 bp of overlap, using the “bedtools merge -d 0” command, into a master peak list for downstream analysis. This “pooling and merging” strategy can potentially create broader peaks that do not accurately reflect the true positions of epigenetic modifications, particularly when many cell types with strong signals are present. Therefore, we recommend the use of master cCRE lists generated by data consortia project such as ENCODE [6], or generation of a master peak list by using peak calling methods that are designed to handle datasets across multiple cell types, such as S3V2-IDEAS package’s Intensity State mode [38]. In this study, we used a cCRE list generated by the S3V2-IDEAS package’s Intensity State mode with default settings.

### Determining the number of clusters for different clustering methods

For K-means, Mclust, and hierarchical clustering followed by branch trimming using “cutree” function, we set the number of clusters (K) equal to 69. This number matches the number of clusters from Snapshot, which was automatically determined by the distribution of the number of cCREs per IS.

### Evaluating reproducibility of clustering results by *adjusted random index*

To evaluate the reproducibility of the clustering results, we repeatedly clustered the same data after adding random noise 5 times for each clustering method, using different random noise each time. We computed the random noise as a set of uniformly distributed random numbers ranging from -0.1 to 0.1. We then calculated the pairwise adjusted random index (ARI) of the 5 clustering results [39, 40]. The ARI is a widely used measure of the consistency between two sets of clustering results. When ARI equals 1, it means two sets of clustering results are exactly the same. When ARI is close to 0, it means two sets of clustering results are equivalent to two sets of randomly ordered labels. To reduce the computational time, we perform this analysis in randomly selected subsets of row from the original data matrix for Hclust and Mclust analysis.

## Results

### Clustering and visualizing *cCREs* in the *hematopoietic system*

We developed the Snapshot package to help find and analyze informative groups of cCREs in blood cells, using resources from the VISION project [28–30, 41]. In this report, we use a set of cCREs identified in human blood cell types [42]. We first called peaks on the chromatin accessibility data in 13 hematopoietic primary cell types (Fig. 1D). Then, we downloaded from the VISION project website [43] the list of 200,342 human hematopoietic cCREs, which were determined on a larger number of cell types and cell lines. The subset of 83,701 cCREs that intersect with at least one peak in these 13 hematopoietic cell types was used for analysis in Snapshot.

We treat each of the 83,701 genomic locations as a cCRE. To find clusters of cCREs, each cCRE is labeled with a 13-digit binarized index, in which each digit corresponds to the presence (1) or absence (0) of a peak call for that cCRE in each of the 13 hematopoietic cell types (step 1, Fig. 1A). Grouping cCREs by their indices produced 1,806 ISs, with each IS containing cCREs with identical indices (step2, Fig. 1B)). Most ISs only contains a few cCREs (Fig. 2A). By default, Snapshot filtered (temporarily removed) 1,738 ISs (step3, Fig. 1B) and retained 68 abundant ISs that contained more than 173 cCREs (red dashed line in Fig. 2A). For the cCREs in the filtered ISs, about 85% (17,024 cCREs) of them were then re-classified into one of the abundant ISs in the rescuing step (step4, Fig. 1C), which is based on matching the profile of the cCRE to the distribution of signals for each IS. The filtering and rescue steps increased the sizes of ISs that passed the abundance threshold (Fig. 2B). The remaining cCREs, specifically those with a re-classification posterior probability less than 0.5, were clustered into one additional null class IS. To improve the interpretability and simplify the results, we employed a second-round clustering procedure to merge the 69 ISs (Fig. 1D) into 19 Meta-ISs (Fig. 1E) based on their average signal across all 13 cell types.

We next compared the results from Snapshot to those from three existing methods, namely K-means clustering, hierarchical clustering (Hclust), and Gaussian Mixture Modeling for Model-Based Clustering (Mclust), in terms of the interpretability, comprehensiveness, and reproducibility.

### Comparison of clusters by interpretability

The comparison of interpretability is based on the patterns of binary peak calls for clustered cCREs across cell types. We constructed two dimensional (2D) maps for the clustering results from each method, with the binary peak calls displayed for each cCRE across cell types (Fig. 3A–D). For the y-axis in the map of Snapshot results, we sorted the ISs by the indices of ISs along a linearized representation of the cell differentiation tree (Fig. 3A). For maps of results of other clustering methods, the cCREs were ordered by using their cluster labels (K-means and Mclust) or cluster output orders (Hclust) (Fig. 3B–D).

The 2D map of Snapshot results shows the ISs and the corresponding cCRE accessibility history during the cell differentiation. For example, a large group of cCREs are in accessible chromatin in common myeloid progenitors (CMP), with a subset remaining accessible in megakaryocytic erythroid progenitors (MEP), and a smaller subset that remain accessible during erythroid (ERY) maturation (red box in Fig. 3A). Illustrating the ability of Snapshot to find meaningful but small ISs, the IS with cCREs that are only accessible in T-CD8 cells can be clearly identified (purple box in Fig. 3A). The maps of results of the other methods show many interpretable clusters, such as those specific to a particular cell type or lineage, but they are mixed with a large number of less interpretable clusters (Fig. 3B–D). The order of clusters from the other methods cannot be easily sorted by the same approach as used in Snapshot, because their clustering space is continuous while the cell type space is categorical. Thus, even if their clustering results captured patterns of accessibility across cell types similar to those from Snapshot, the organization of these clusters in the 2D map makes it difficult to distinguish the more meaningful clusters from other clusters that may be less informative.

We also compared the 2D representation of the clustering results quantitatively by Shannon Entropy (SE), making the underlying assumption that clustering patterns with lower entropy, and hence less randomness, may represent better interpretability. The specific procedures for calculating SE are described in the Implementation section. Computing the SE using a series of sliding 2D windows over the binarized clustering maps (Fig. 4) gave consistently lower SE values for Snapshot results compared to the results of other methods (Fig. 3E). The lower SE values, and inferred greater interpretability, for Snapshot were observed robustly across a series of settings varying the number of adjacent cCREs or number of cell types included in the 2D window used in the measurement (Fig. 3E).

### Snapshot index identifies detailed *cCRE* patterns within other clustering results

**Fig. 5:**
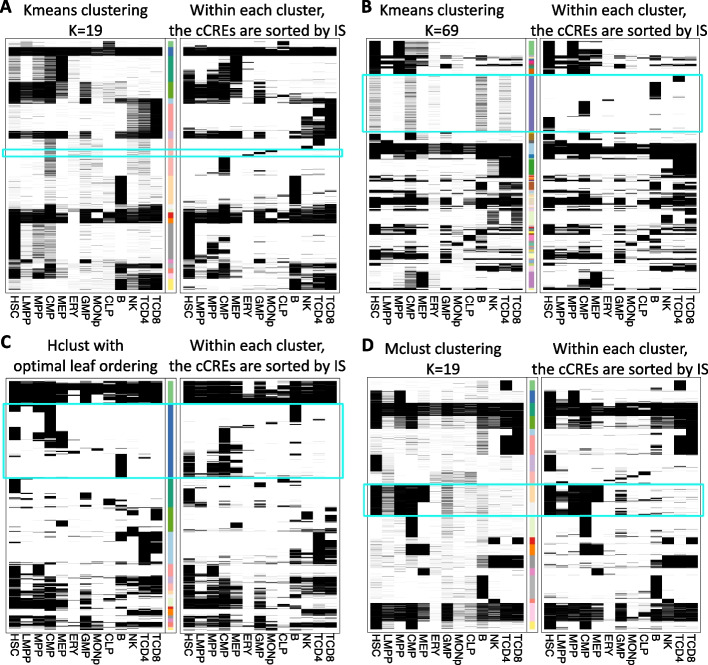
The results of re-sorting by Snapshot indices of cCREs in clusters resulting from four existing methods: **A** K-means clustering (K = 19);** B** K-means clustering (K = 69);** C** Hierarchical clustering (K = 19); **D** Mclust clustering (K = 19). The left-side heatmap displays the clustering results with the cCREs within each cluster ordered based on the default outputs of each method. The cluster labels are represented by the bars with different colors, where each color indicates a unique cluster label. In the right side heatmap, the cCREs within each cluster are reordered based on the indices generated by Snapshot

The next evaluation is based on the reasoning that if Snapshot is better able to find interpretable clusters, then it should be able to uncover finer-resolution sub-clusters within the results generated by other commonly used clustering methods, especially in the larger clusters. To investigate this hypothesis, we constructed pairs of heatmaps for the different clustering methods (Fig. 5A–D). In the left-side heatmap of each pair, the cCREs within each cluster were ordered based on the default output of each clustering methods, while in the right-side heatmap, the cCREs within each cluster were reordered by their Snapshot indices. For K-means (K = 19 and 69), Hclust (K = 19), and Mclust (K = 19), the clusters reordered by the Snapshot indices show more detailed and organized patterns. For example, in one cluster from K-means (K = 19) reordered by the Snapshot indices, the cCREs that are specifically activated in MEP, ERY, and granulocyte/macrophage progenitor (GMP) cells become identifiable (Fig. 5A cyan box). Similar improvements after the Snapshot index reordering are highlighted with cyan boxes in other heatmaps. The results from Hclust can identify some small but distinct clusters. Its output cluster labels, however, are decided by the cutting point of the hierarchical tree. As a result, those distinct clusters are merged into larger ones (Fig. 5C cyan box). Using K-means (K = 69) did reveal some detailed patterns (Fig. 5B), showing that K-means with a sufficiently large number of clusters can identify rare clusters. However, it is challenging to determine the appropriate number of clusters needed for K-means to reveal the rare clusters.

### Snapshot identifies highly reproducible *cCRE patterns*

**Fig. 6:**
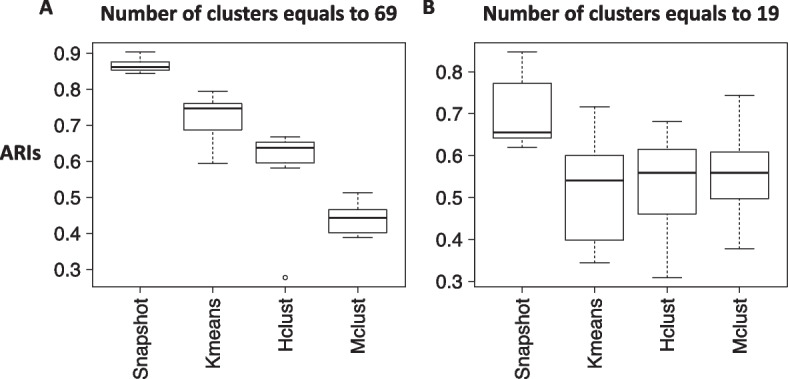
Comparing the robustness of four Clustering Methods after adding random noise to the signal matrix. The robustness is quantified by pairwise Adjusted Rand Index (ARI) between cluster labels generated by 5 rounds of clustering runs. The number of output clusters used in all methods are equal to 69 (panel **A**) and 19 (panel **B**)

Some level of technical noise is inevitable in high throughput sequencing data, and thus, clustering methods that are robust to technical noise are valuable for identifying reproducible and reliable patterns in the data. To evaluate the reproducibility of the clustering results, we repeatedly clustered the same data after adding different random noises (uniformly distributed from -0.1 to 0.1) for 5 times for each clustering method. We then calculated the pairwise Adjusted Rand Index (ARI) between different sets of clustering results for each method [39]. The results of the Snapshot method had significantly higher overall ARIs (Wilcoxon test using the wilcox.test function in R, p-value = 5.4e-6 (K = 69) and 2.4e-4 (K = 19)) than those from other methods (Fig. 6), which indicates the results are more robust to the addition of simulated noises and thus should be more reproducible than other examined methods when analyzing real data.

### Validate the biological significance of Meta-IS through orthogonal data

**Fig. 7:**
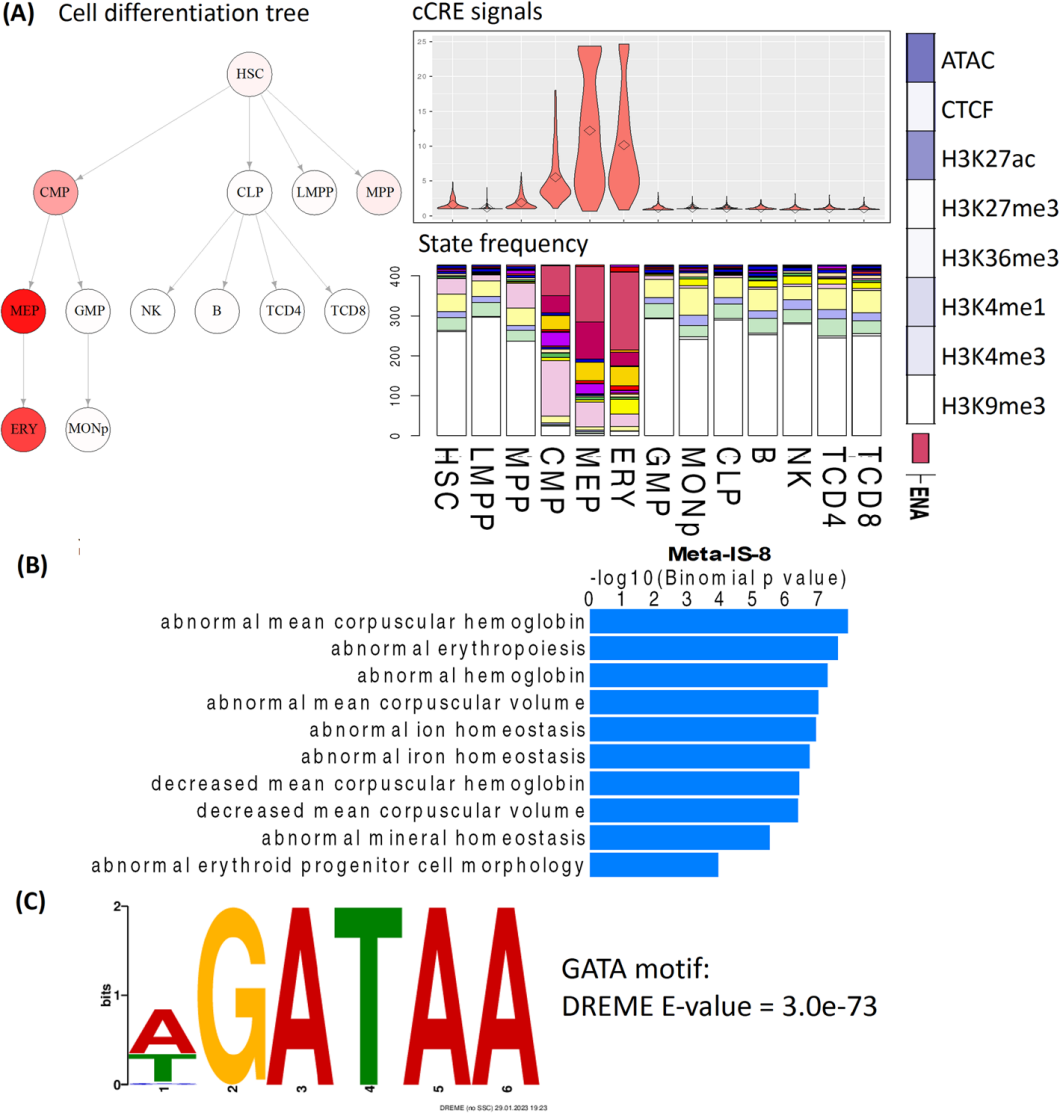
The Snapshot visualizations for Meta-IS-8. **A** The hematopoietic cell differentiation tree colored by average chromatin accessibility signal of the cCREs in Meta-IS-8 (left). The distributions of those accessibility signals in each cell type are shown as violin plots (right top). The bar plot (right bottom) displays the proportion of each epigenetic state annotation of the cCREs in this Meta-IS. The single column heat map (far right) shows the emission frequencies of epigenetic features from the dominant epigenetic state labeled ENA (enhancer, nuclease accessible, activated). **B** The mouse phenotype terms in GREAT analysis that are significantly enriched in Meta-IS-8. **C** The most significantly enriched TF binding motif in Meta-IS-8 identified by DREME analysis in the GATA motif (E-value = 3e-73)

The output of Snapshot can reveal specific ISs and Meta-ISs of interest that may be missed by other clustering methods. For example, Meta-IS-8, a cluster containing 427 cCREs (Fig. 1F), contains cCREs that may be involved in erythroid gene activation, but it is only rarely revealed in 100 rounds of K-means clustering (Fig. 1G). The Snapshot clustering of chromatin accessibility peaks indicated that the cCREs in Meta-IS-8 are actuated (called as peaks) primarily in the progenitor and mature erythroid cells (Fig. 1E), indicating a role in erythroid gene regulation. This inference is supported by orthogonal evidence, and the visualization output from Snapshot gives insight into the epigenetic transitions of these cCREs during differentiation (Fig. 7). Specifically, the nuclease accessibility of cCREs in this Meta-IS gradually increased from the progenitor cells to the erythroblasts, and the number of cCREs annotated with an active epigenetic state increased as cells differentiate along the path from CMP to ERY (Fig. 7A). The epigenetic state annotation was generated by the IDEAS 2D genome segmentation method [11] in the VISION project [42]. These observations suggested a hypothesis that these cCREs may be critical for erythroid differentiation. This hypothesis predicted that the functional ontology terms of the genes regulated by this set of cCREs should be enriched for erythropoiesis, and that the cCREs would be enriched in DNA binding motifs for erythroid transcription factors. To test the hypothesis, we examined the Mouse Phenotype terms of genes associated with these regions using GREAT [44], and we confirmed that the cCREs in Meta-IS-8 were significantly associated with hemoglobin and erythroid related terms (Fig. 7B). Furthermore, the most significantly enriched transcription factor binding motifs (from DREME) [45, 46] were those for the GATA transcription factor family (Fig. 7C). It is known that two GATA factors, GATA1 and GATA2, are critically important for erythroid cell differentiation [47].

**Fig. 8:**
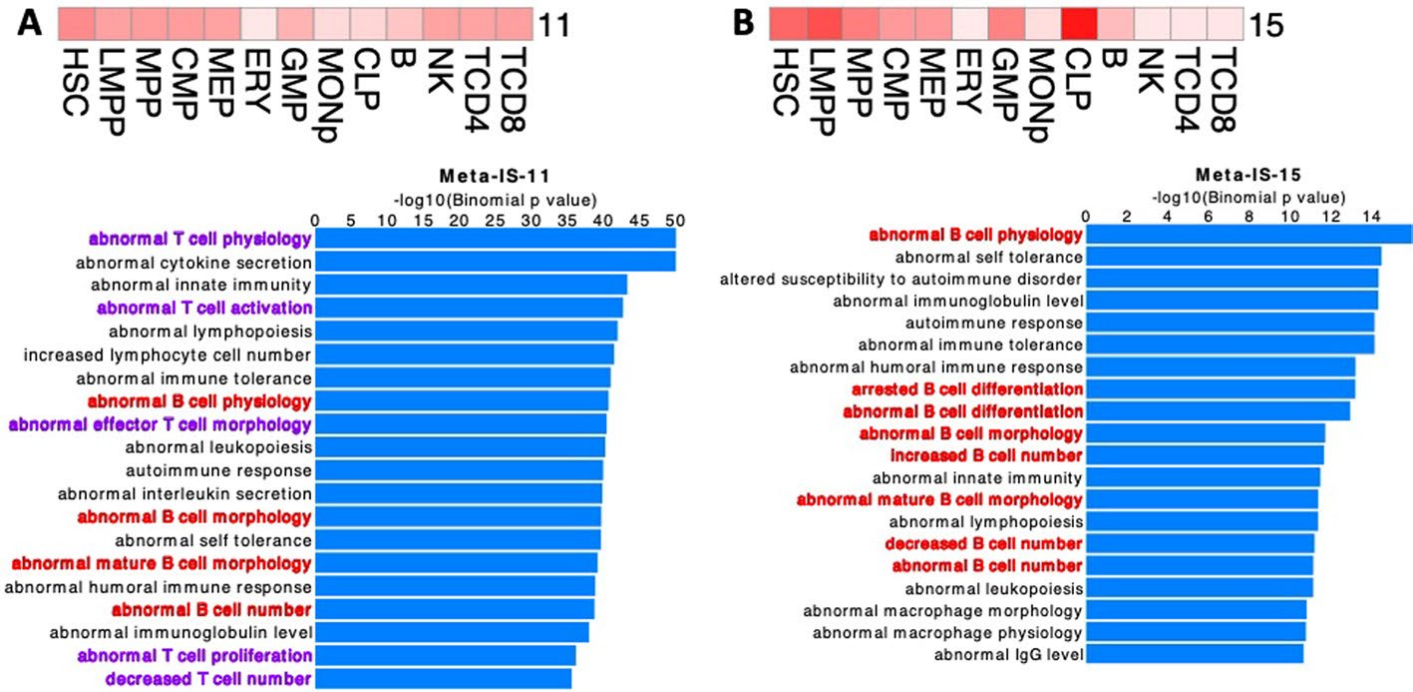
Chromatin accessibility and functional term enrichments for cCREs in Meta-IS-11(**A**) and Meta-IS-15 (**B**). The top heatmap in each panel displays the average chromatin accessibility signals of the cCREs in the Meta-IS cluster across 13 cell types. The bottom part of each panel presents mouse phenotype terms and their corresponding -log10 p-values based on binomial background model. In the GREAT analysis for enrichment of function-related terms, the proximal regions are defined as TSS -5 kb to + 1 kb, and the distal regions are set to be proximal regions ± 100 kb

Meta-IS-11 and Meta-IS-15 are additional examples of metaclusters discovered by Snapshot but not found frequently by K-means clustering (Fig. 1G). The cCREs in both of these metaclusters are actuated in stem and progenitor cells, but they differ in their actuation in lymphocytes (Fig. 8A and B). Specifically, the cCREs in Meta-IS-11 are also actuated in natural killer (NK), CD4 + T, and CD8 + T cells, but weakly in B cells. In contrast, the cCREs in Meta-IS-15 are actuated in B cells and the common lymphoid progenitors (CLP), but not in NK, CD4 + T, and CD8 + T cells. These different patterns of cCRE actuation suggested the hypothesis that the cCREs in the two metaclusters may be involved in regulating genes needed in the different branches of lymphopoiesis. To test this hypothesis, we used the GREAT tool to find enrichment for mouse phenotype terms for genes associated with the cCREs in each metacluster, which confirmed the hypothesized functional association. The cCREs in both metaclusters showed enrichment for immune-related terms, but those in Meta-IS-11 were associated with several T cell terms and B cell terms whereas those in Meta-IS-15 were mainly associated with B cell related terms (Fig. 8).

These findings illustrate the effectiveness of Snapshot in identifying biologically meaningful clusters that may be missed by other commonly used clustering methods.

## Conclusions

The Snapshot package can automatically generate ISs of cCREs or other epigenetic or transcriptomic features in a manner that readily aligns with cellular progression, such as a cell differentiation series. This index-based clustering strategy easily reveals all distinct clusters of lineage-specific or stage-specific epigenetic events without requiring predetermined parameters such as the number of clusters. While the index-based approach can produce a large number of clusters initially, one can leverage the imbalance in the sizes of clusters to obtain a manageable number of clusters after the filtering and rescuing procedures. The number of groups can be reduced further by an additional round of clustering to merge ISs into Meta-ISs, which can give a more easily interpretable final set of metaclusters. In addition, the rescue step in Snapshot borrows information across multiple cell types to correct potential peak calling errors, which can help to improve the accuracy of clustering based in epigenetic features across different cell types or conditions [48]. While we have demonstrated the Snapshot package for analyzing cCREs across blood cell differentiation, it can be used to study any progression of cell types, such as those responding to hormones or signaling factors or those along a developmental series. Larger sets of epigenomic data are allowed in the Snapshot package. Exploring the utility of Snapshot for much larger numbers of datasets, especially examining the metaclusters of ISs, could be a productive future direction. Furthermore, the clustering and visualizations from Snapshot can reveal groups of elements that may play roles in key transitions in the transcriptome and epigenome during the cellular progression being studied. All these features together make Snapshot a package that can improve the interpretability, comprehensiveness, and robustness for clustering and interpreting the cCREs or other epigenetic events across multiple cell types in a system.

## Data Availability

The Snapshot package is available at GitHub (https://github.com/guanjue/Snapshot [1]) with MIT License. The main part of Snapshot is written in python with some R scripts for visualization. For running Snapshot, we provided a conda environment that can be deployed in both MacOS and Linux operating system. Files for raw signals, p-value converted signals, and signals from S3norm are available both for download and for viewing from the VISION website (http://usevision.org [43]). The list of links for the files used in this paper can be found in this link (https://github.com/guanjue/snapshot/blob/main/test_data/Snapshot_paper.all.file.links.txt [49]).

## Abbreviations

VISION: ValIdated systematic integration of hematopoietic epigenomes
cCRE: Candidate cis-regulatory-element
IS: Index-set
Meta-IS: Meta-Index Set
MVN: Multivariate Gaussian distribution
NB: Negative binomial
QDA: Quadratic discriminant analysis
Hclust: Hierarchical clustering
Mclust: Model-based clustering
SE: Shannon entropy
ARI: Adjusted random index
SD: Standard deviation
GMP: Granulocyte/macrophage progenitor
CMP: Common myeloid progenitors
MEP: Megakaryocytic erythroid progenitors
ERY: Erythroid
MK: Megakaryocytic

## Declarations

### Other notes

Proofreading for some sentences was done using the ChatGPT model by inputting the phrase 'improve this sentence: ' followed by the sentence into the ChatGPT input box. The output was manually reviewed to ensure it accurately conveyed the original meaning, and any newly identified typographical errors were corrected. All text was subsequently edited by the authors.
